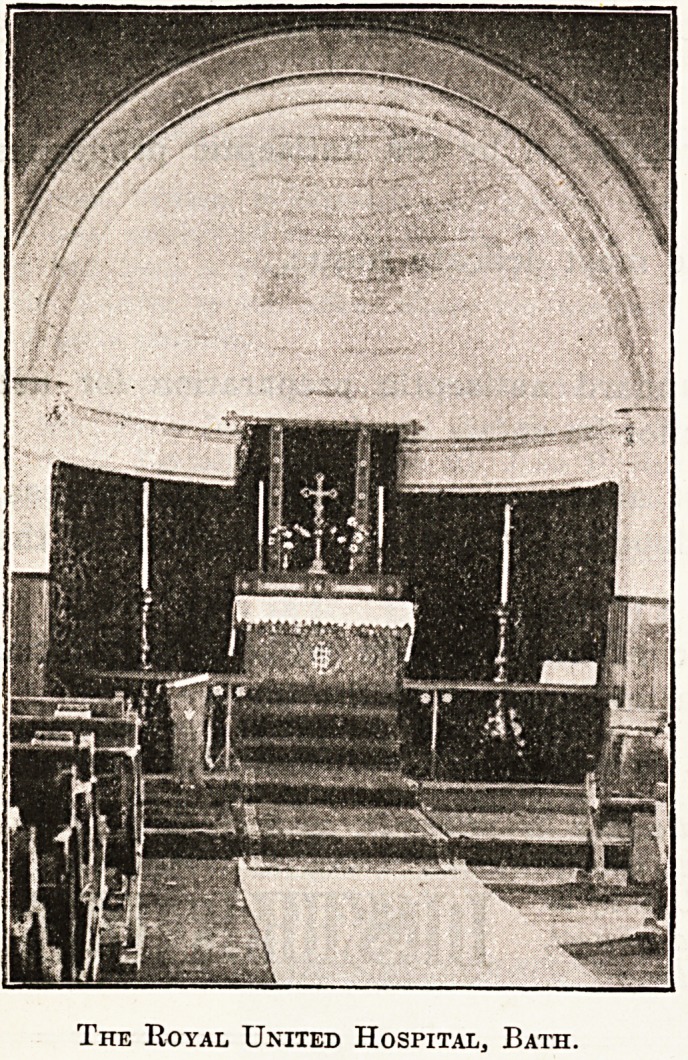# Some Provincial Hospital Chapels

**Published:** 1915-01-23

**Authors:** 


### SOME PROVINCIAL HOSPITAL CHAPELS.

Royal Hampshire County Hospital.

So much interest was aroused by an article on "Some Chapels of Famous Hospitals" which appeared in The Hospital of April 18, 1914, that a special investigation has been made by the same writer, an experienced chaplain, on the more remarkable of hospital chapels in the provinces.

The Royal Hampshire County Hospital Chapel.

The County Hospital at Winchester, to begin with, which opened on St. Luke's Day, 1736, and was the first of its kind in any part of the kingdom, except in London, does not appear at that time to have possessed any chapel, but the clergy of Winchester were "desired to attend by weekly rotation to visit the sick and to read prayer every day in the wards, and to give Communion at proper times." When the hospital was removed in 1868 to a new site and the new building erected from the designs of Mr. Butterfield, and thenceforward known as the Royal Hampshire County Hospital "by command of Queen Victoria," the chapel was a prominent feature, and was dedicated on June 30, 1868, by Bishop Moberly, of Salisbury. The building, which altogether cost some £1,500, is of an ornamental design, and possesses ample accommodation for the needs of the hospital. The chapel might almost be described as a memorial to the late Warden Barton, of Winchester College, since nearly half the necessary funds were provided by friends who desired to erect to his memory a suitable memorial expressive of their love and respect; among the subscribers are to be found the names of Charlotte Yonge and John Keble. It is interesting to note that by the statutes the Holy Communion has to be celebrated every Sunday, Christmas Day, and Ascension Day.

Royal United Hospital, Bath.

Reverence and simplicity is the keynote of the chapel of the Royal United Hospital, Bath. The building, which is of stone, is rectangular in shape, with a small domed sanctuary, the carved altar, ornaments, and furniture of which are at once suitable and impressive. There are two memorial stained-glass windows, one bearing the inscription, "In memory of Nurse Evans, called to rest April 12, 1896," the other, "In loving memory of Kathleen (Kitty) Thomas—'Sister Wales'—who fell asleep on Thursday, March 6, 1902," while a brass tablet commemorates the founder of the chapel, the Rev. Edward Handley, who was also president of the hospital from 1890-1904, and who died on February 1, 1904. A small one-manual organ is placed at the west end of the chapel,

The Royal United Hospital, Bath.

and the accommodation is ample for the needs of the institution. The Rev. Prebendary S. A. Boyd is the Chaplain to the institution.

General Hospital, Birmingham.

Centrally placed on the first floor of the hospital, the chapel of the Birmingham General Hospital is fitted with oak benches, and has an oak and plaster ceiling with carved oak frieze. The walls of the building are lined with marble and Derbyshire alabaster, and the pulpit and altar-rails are of the same material. The west windows, which are of stained glass, and the pulpit are a memorial to the late Mr. T. H. Bartleet, F.R.C.S.; one of the windows on the south side is in memory of the late Mr. Robert Jolly, M.D., F.R.C.S., while a third commemorates the services rendered to the hospital by Mr. Edward Malins, M.D., F.R.C.P., who resigned in January 1903. Two cast bronze tablets on the walls are dedicated to the memory of a former matron and assistant surgeon, the inscription on one of which is as follows: "Mary Esther Jones, Matron of the General Hospital, 1898-1908. Died June 6, 1909. This tablet was erected in affectionate remembrance by many members of the Nursing Staff."

Another tablet, which was "erected by members of the Board of Management and his medical and surgical colleagues," reads as follows: "In memory of Frederic Victor Milward, B.A., M.B. Cantab., F.R.C.S., Assistant Surgeon to the Hospital. Born July 20, 1869. Died March 31, 1910. And who was for 11 years an Officer of the Hospital." The Rev. M. P. Hellier is the Chaplain to the institution.

(*To be continued.*)

## Figures and Tables

**Figure f1:**
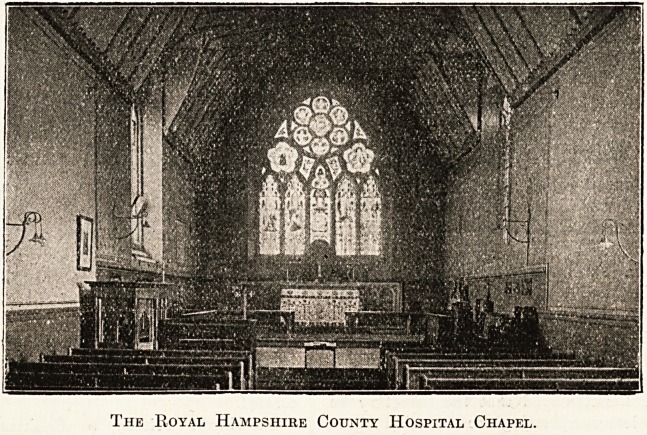


**Figure f2:**